# Which exercise modality improves vascular function most in overweight or obese adults? A Bayesian network meta-analysis of randomized controlled trials

**DOI:** 10.3389/fphys.2026.1806291

**Published:** 2026-08-07

**Authors:** Xiang Yu, Yuanfeng Liu, Canbo Wang, Zhiyuan Tan

**Affiliations:** 1 Party Committee Office & Principal’s Office, Chengdu University of Technology, Chengdu, Sichuan, China; 2 Postdoctoral Research Station for Management Science and Engineering, Xi’an University of Technology, Xi’an, Shaanxi, China; 3 Sports Institute, Chengdu University of Technology, Chengdu, Sichuan, China; 4 Faculty of Sport and Physical Education, University of Belgrade, Belgrade, Serbia

**Keywords:** exercise training, flow-mediated dilation, network meta-analysis, obesity, vascular function

## Abstract

**Background:**

Exercise is recommended for adults with overweight or obesity, but the comparative vascular effects of different exercise modalities remain uncertain.

**Methods:**

We conducted a systematic review and Bayesian network meta-analysis of randomized controlled trials enrolling adults with overweight or obesity. PubMed, Web of Science, the Cochrane Library, and EBSCO SPORTDiscus were searched from inception to 31 May 2025, supplemented by Google Scholar and reference screening. Eligible interventions lasted at least 4 weeks and were classified as continuous endurance training (CET), resistance training (RT), interval training (INT), combined aerobic-resistance training (CT), or hybrid/mixed-modality programs (HYB). The primary outcome was flow-mediated dilation (FMD); secondary outcomes were pulse wave velocity (PWV) and carotid intima-media thickness (CIMT). Risk of bias was assessed using RoB 2 and certainty using CINeMA.

**Results:**

Fifty-one randomized trials (2,638 participants) were included. Exercise improved FMD (standardized mean difference [SMD], 0.99; 95% CI, 0.69 to 1.29), reduced PWV (SMD, -0.31; 95% CI, -0.44 to -0.18), and reduced CIMT (SMD, -0.20; 95% CI, -0.36 to -0.05). In descriptive treatment-versus-control rankings, HYB had the largest FMD estimate, INT had the largest PWV reduction, and CET/CT had the largest CIMT reductions; however, these rankings were imprecise and certainty was low. FMD showed substantial heterogeneity (I2 = 87.1%). Sensitivity analyses excluding high-risk-of-bias studies and specialized cardiac or renal populations did not materially change the direction of findings.

**Conclusions:**

Exercise interventions are associated with favorable vascular changes in adults with overweight or obesity. Apparent modality-specific differences should be interpreted as exploratory rather than definitive because confidence in the evidence was low and several comparisons were sparse or heterogeneous.

**Systematic Review Registration:**

https://www.crd.york.ac.uk/PROSPERO/, identifier CRD420251066443.

## Introduction

Overweight and obesity remain major public-health challenges and are closely linked to cardiometabolic disease and atherosclerotic cardiovascular disease (Van Gaal et al., 2006; Wyatt et al., 2006; Esser et al., 2014; Williams et al., 2015; Chooi et al., 2019; Bays et al., 2024; Rubino et al., 2025). Excess adiposity promotes endothelial dysfunction, dyslipidemia, insulin resistance, oxidative stress, and chronic low-grade inflammation, all of which accelerate atherosclerotic initiation and progression (Libby and Theroux, 2005; Després and Lemieux, 2006; Falk, 2006; Krysiak et al., 2012; Amato and Giordano, 2014; Longo et al., 2019; Koenen et al., 2021; Libby, 2021; D’Oria et al., 2022; Frąk et al., 2022; Jebari-Benslaiman et al., 2022). Because vascular dysfunction can precede overt cardiovascular events, interventions that improve vascular structure and function are clinically relevant in this population.

Exercise is a first-line non-pharmacological strategy for obesity management and cardiovascular prevention (Higuera-Hernández et al., 2018). Regular physical activity can reduce total and visceral adiposity, improve lipid and glucose metabolism, lower systemic inflammation, and enhance nitric oxide-mediated endothelial function (Holloszy, 2005; Kasapis and Thompson, 2005; Duncker and Bache, 2008; Escalante et al., 2012; Lee and Lee, 2021; Mućka et al., 2022). However, exercise prescriptions vary widely across studies, including continuous aerobic training, resistance training, interval training, combined aerobic-resistance protocols, and more heterogeneous hybrid or mixed-modality programs.

Several randomized trials have assessed exercise effects on flow-mediated dilation (FMD), pulse wave velocity (PWV), and carotid intima-media thickness (CIMT), which represent complementary vascular domains: endothelial function, arterial stiffness, and structural remodeling, respectively (Ho et al., 2012; Bruno et al., 2014; Ramírez-Vélez et al., 2016; Park et al., 2017). Existing reviews have often focused on single outcomes or broad cardiometabolic risk factors and have not fully compared exercise modalities across multiple vascular endpoints in adults with overweight or obesity.

Therefore, we conducted a systematic review and Bayesian network meta-analysis of randomized controlled trials to compare exercise modalities for improving FMD, PWV, and CIMT in adults with overweight or obesity. Given expected clinical and methodological heterogeneity, modality rankings were interpreted alongside certainty of evidence, heterogeneity, small-study effects, subgroup analyses, and sensitivity analyses rather than as stand-alone proof of superiority.

## Methods

### Protocol and reporting

This review followed PRISMA guidance and the PRISMA extension for network meta-analyses (Moher et al., 2009; Hutton et al., 2015). The protocol was prospectively registered in PROSPERO (CRD420251066443).

### Search strategy

PubMed, Web of Science, the Cochrane Library, and EBSCO SPORTDiscus were searched from inception to 31 May 2025. Search terms combined concepts for overweight/obesity, adults, exercise modalities, and vascular or atherosclerosis-related outcomes. Google Scholar and reference lists of relevant articles were screened manually. The complete database strategies are provided in **Supplementary Table 1**.

### Eligibility criteria

Eligibility was defined using PICOS. Populations were adults aged 18–65 years with overweight or obesity, defined by body mass index of at least 25 kg/m2 or by the original trial definition. Disease-specific cohorts were eligible only when the cohort also satisfied the overweight/obesity criterion. Eligible interventions were structured exercise programs lasting at least 4 weeks. Comparators were usual lifestyle, no-exercise control, conventional therapy, or standard care. Outcomes were pre- and post-intervention data for FMD, PWV, or CIMT. Only published randomized controlled trials with sufficient data for quantitative synthesis were included.

### Exercise modality classification

Exercise arms were classified *a priori* according to the dominant prescribed training stimulus. CET referred to continuous rhythmic aerobic or endurance exercise without repeated high-intensity intervals or a prescribed resistance component. RT referred to structured strength or resistance exercise. INT referred to high-intensity interval, sprint interval, or aerobic interval protocols. CT referred to programs that separately prescribed continuous aerobic/endurance and resistance components. HYB referred to mixed, recreational, technology-assisted, vibration, sedentary-behavior-reduction, rehabilitation-platform, or otherwise multimodal interventions that could not be decomposed into clearly prescribed CET plus RT components. Multi-arm trials were classified at the arm level.

### Data extraction and risk of bias

Two reviewers independently extracted study characteristics, country or region, sample size, intervention type, intervention duration and frequency, outcomes, and adverse events. Outcome data were extracted as mean and SD values and synthesized primarily as change scores (post-intervention minus baseline), because baseline vascular function differed across small trials and post-only values may be sensitive to baseline imbalance. Risk of bias was assessed with RoB 2. Certainty of network evidence was evaluated using CINeMA across within-study bias, reporting bias, indirectness, imprecision, heterogeneity, and incoherence (Nikolakopoulou et al., 2020).

### Statistical analysis

Continuous outcomes were synthesized as standardized mean differences (SMDs) with 95% confidence intervals (CIs). The primary analysis used pre–post change scores rather than post-intervention values because baseline vascular function differed across trials and small sample sizes made post-only estimates vulnerable to baseline imbalance. When SDs of change were unavailable, they were derived from reported statistics or estimated using standard assumptions, with the influence of these assumptions examined in sensitivity analyses. A Bayesian multilevel random-effects model was fitted using the brms package in R. Intervention arms were nested within studies, allowing heterogeneity to be estimated at the between-study level (τ_study) and the within-study, between-arm level (τ_arm). Weakly informative Normal(0, 1) priors were assigned to fixed effects, and half-Cauchy(0, 1) priors were assigned to random-effect standard deviations. The model used eight chains with 3,000 warm-up and 3,000 sampling iterations per chain. Convergence and sampling adequacy were assessed using R-hat values, effective sample sizes, divergent transitions, and posterior predictive checks. Bayesian estimates are reported as posterior means with 95% credible intervals (CrIs).To describe the intervention network and support descriptive ranking, pairwise comparison data were constructed from aggregated study-arm effect sizes, and a random-effects network meta-analysis was performed using the netmeta package. Control conditions, including usual care, no-exercise control, conventional therapy, and health education, were used as the common reference category. Rankings were interpreted alongside CINeMA certainty, heterogeneity, imprecision, and evidence density, and were not treated as stand-alone evidence of superiority. Heterogeneity was quantified using I² and interpreted as low (<50%), moderate (50–75%), or high (>75%). Small-study effects were explored using comparison-adjusted funnel plots. Prespecified subgroup analyses and meta-regression examined sex, region, intervention frequency, intervention duration, and intervention characteristics. Sensitivity analyses examined studies at high risk of bias, specialized cardiac or renal populations, and assumptions regarding missing SDs of change. Network assumptions were assessed before interpreting indirect comparisons. Transitivity was evaluated by comparing prespecified clinical and methodological effect modifiers across intervention nodes, including age, BMI, sex distribution, region, comorbidity profile, intervention duration, exercise frequency, comparator type, and outcome-measurement protocol. Consistency was examined using global and local approaches where closed loops allowed. Because the networks were largely anchored by control comparisons and contained few active-treatment loops, inconsistency tests were considered low powered. Detailed assessments are provided in **Supplementary Tables 4**, **5**. All analyses were conducted in R version 4.5.1 using brms, metafor, and netmeta.

## Results

### Study selection and study characteristics

**Figure 1:**
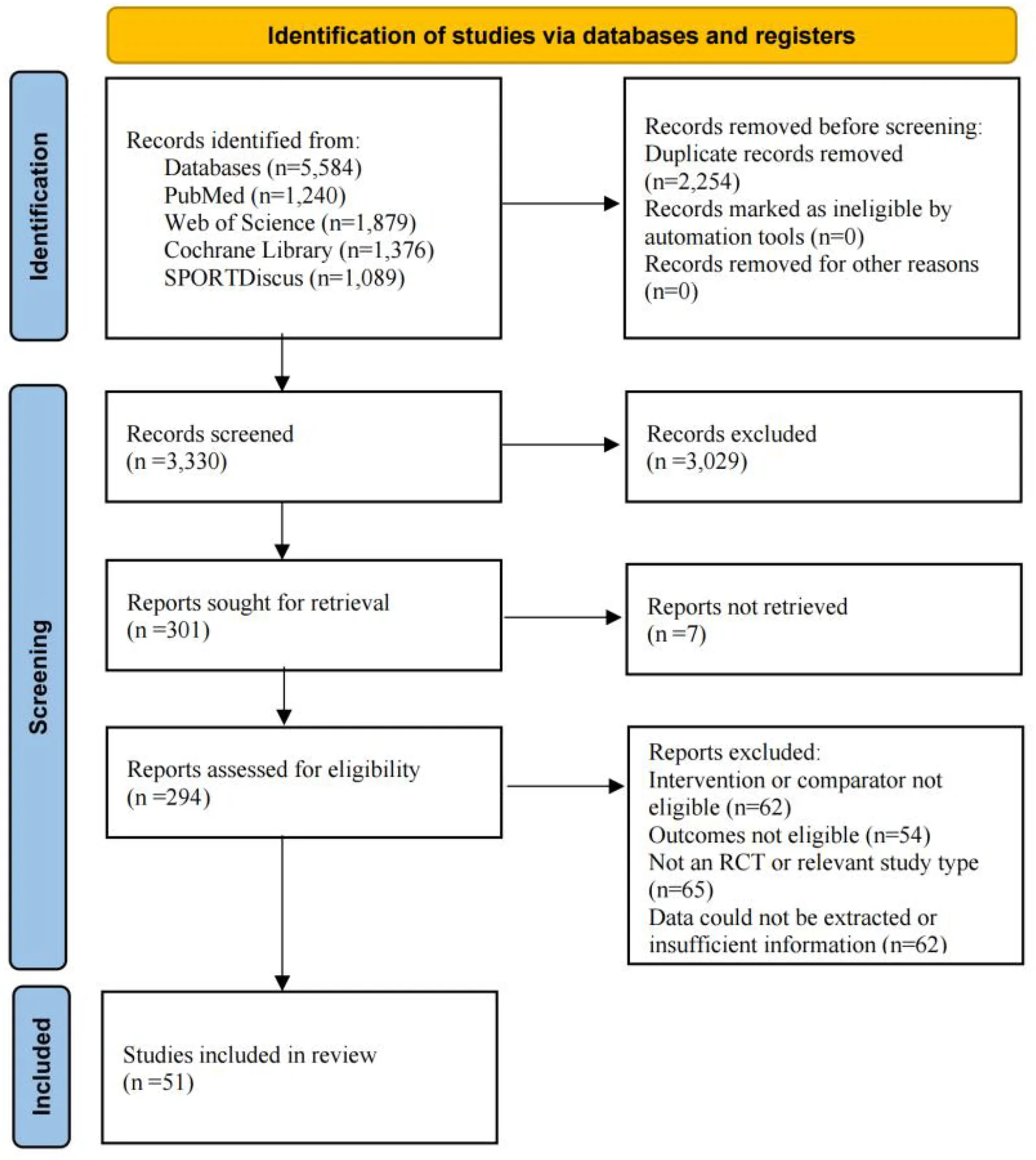
PRISMA flow diagram of study selection.

The search identified 5,584 records. After removal of duplicates, 3,330 records were screened and 294 full-text reports were assessed for eligibility. Fifty-one randomized trials met the inclusion criteria (**Figure 1**). These trials contributed 66 intervention arms and 2,638 participants (1,332 in exercise arms and 1,306 in control arms). Intervention arms comprised CET, RT, INT, CT, and HYB programs. Full trial characteristics are provided in **Supplementary Table 2**.

### Risk of bias and certainty of evidence

All included studies had at least some risk-of-bias concerns, most commonly related to randomization reporting, deviations from intended interventions, or selective reporting. Detailed RoB 2 assessments are shown in **Supplementary Figures 1**, **2**. CINeMA ratings for major control-intervention comparisons were low overall (**Table 1**). Certainty was limited by within-study bias, imprecision for sparse comparisons, and heterogeneity, particularly for CET-related contrasts.

**Table 1:** CINeMA confidence ratings for network estimates.

Comparison	No. studies	Within-study bias	Reporting bias	Indirectness	Imprecision	Heterogeneity	Incoherence	Confidence
Control: CET	28	Some concerns	Low risk	No concerns	No concerns	Major concerns	No concerns	Low
Control: CT	12	Some concerns	Low risk	No concerns	Some concerns	Some concerns	No concerns	Low
Control: HYB	20	Some concerns	Low risk	Some concerns	Major concerns	No concerns	No concerns	Low
Control: INT	17	Some concerns	Low risk	Some concerns	Some concerns	Some concerns	No concerns	Low
Control: RT	20	Some concerns	Low risk	No concerns	Major concerns	No concerns	No concerns	Low

CET, continuous endurance training; CT, combined training; HYB, hybrid/mixed-modality programs; INT, interval training; RT, resistance training.

### Network assumptions

Assessment of transitivity showed that eligibility criteria and control conditions were broadly comparable across major exercise nodes, but important clinical and methodological variability remained, particularly in comorbidity profiles, intervention dose, and evidence density. HYB and INT arms were especially heterogeneous in intervention content and delivery. The networks were predominantly anchored by control comparisons, with few closed loops formed by direct active-treatment comparisons. Formal inconsistency analyses did not identify a major inconsistency signal where assessment was feasible; however, sparse loops limited statistical power. We therefore treated transitivity as plausible but imperfect and interpreted indirect comparisons and rank scores cautiously (**Supplementary Tables 4**, **5**).

### Overall and modality-specific effects

**Figure 2:**
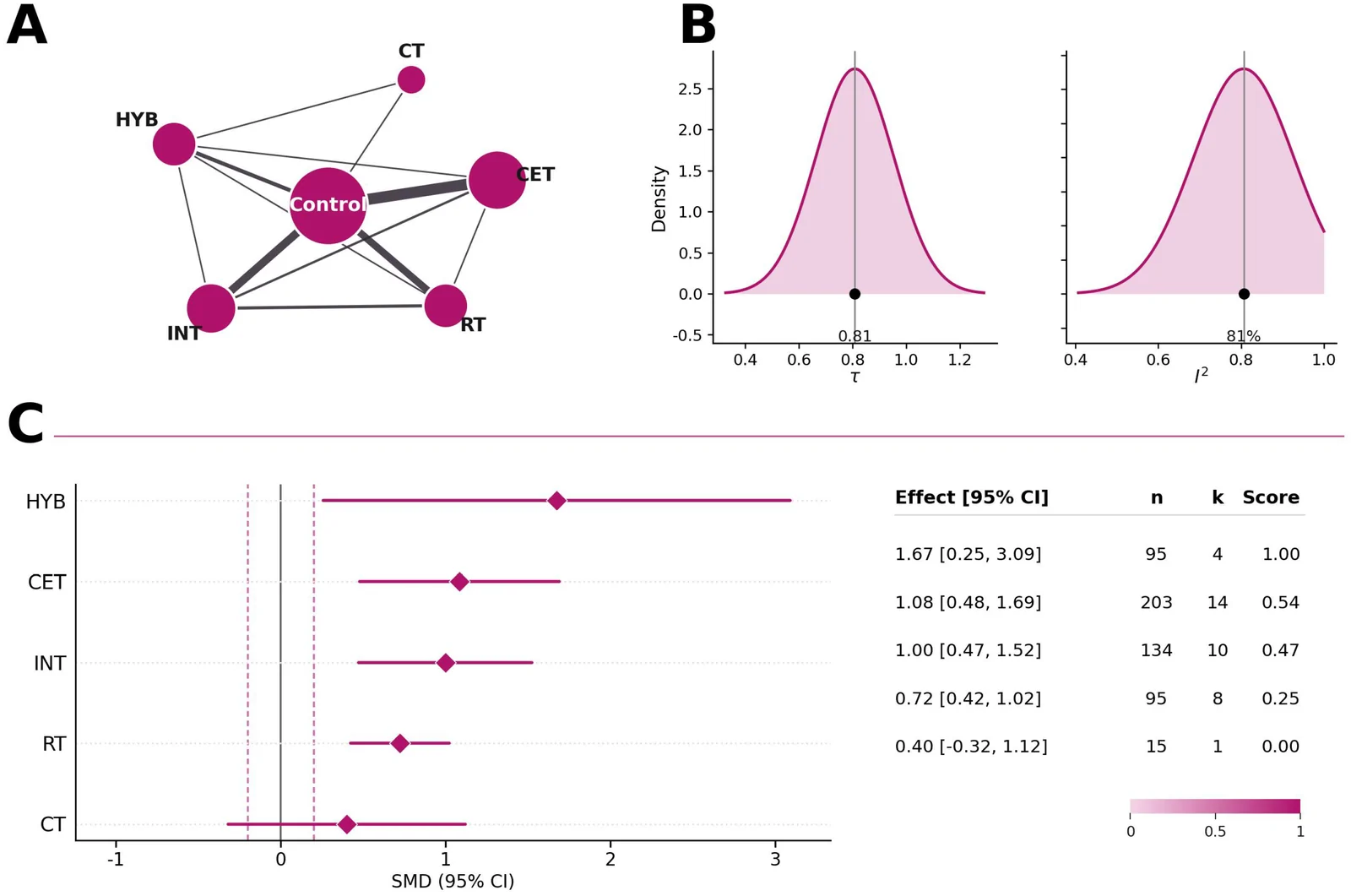
Network structure, heterogeneity distributions, and modality-specific effects for FMD. **(A)** shows the evidence network, **(B)** shows heterogeneity distributions, and **(C)** shows treatment-versus-control estimates and rank scores.

**Figure 3:**
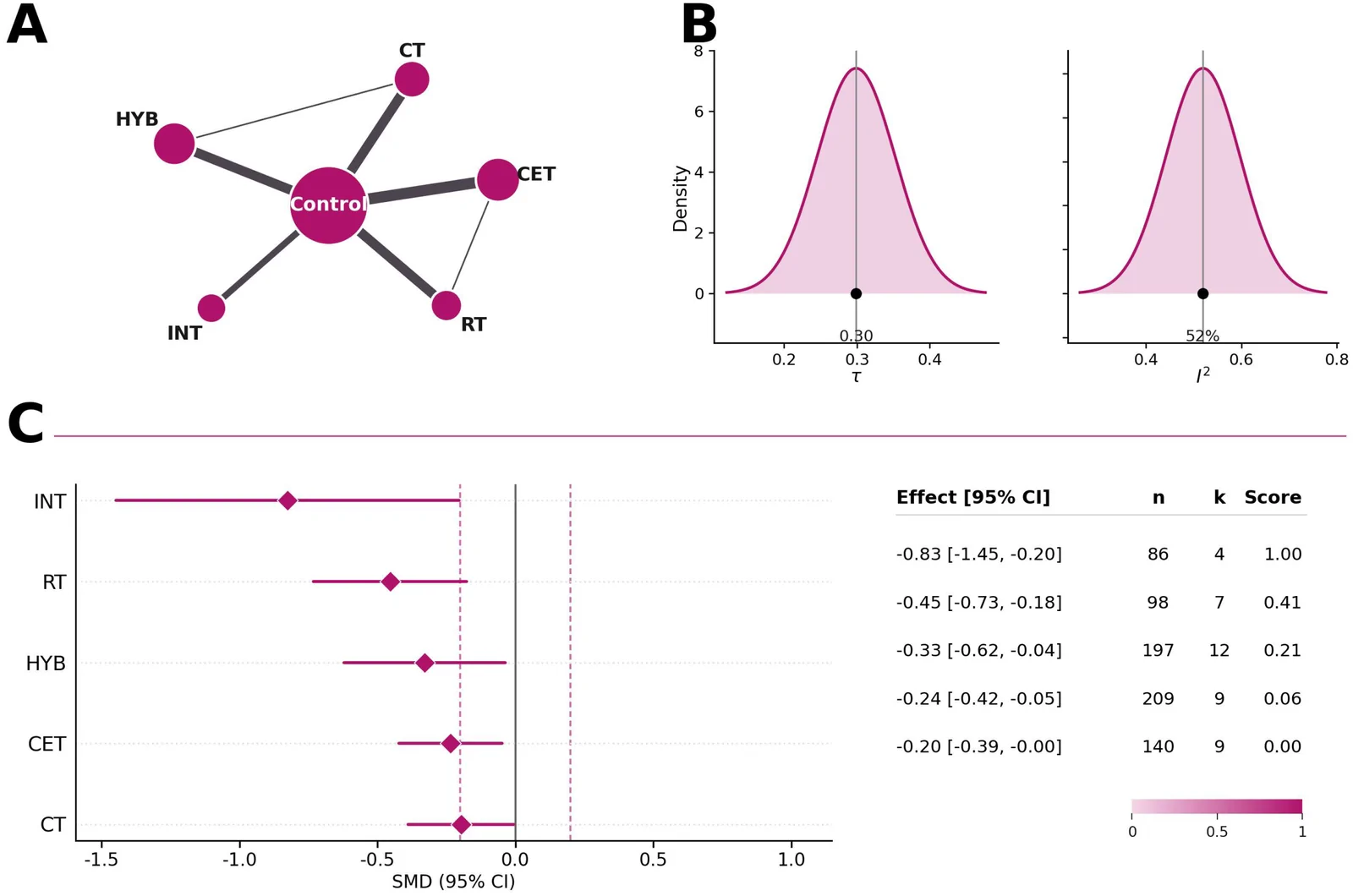
Network structure, heterogeneity distributions, and modality-specific effects for PWV. **(A)** shows the evidence network, **(B)** shows heterogeneity distributions, and **(C)** shows treatment-versus-control estimates and rank scores.

**Figure 4:**
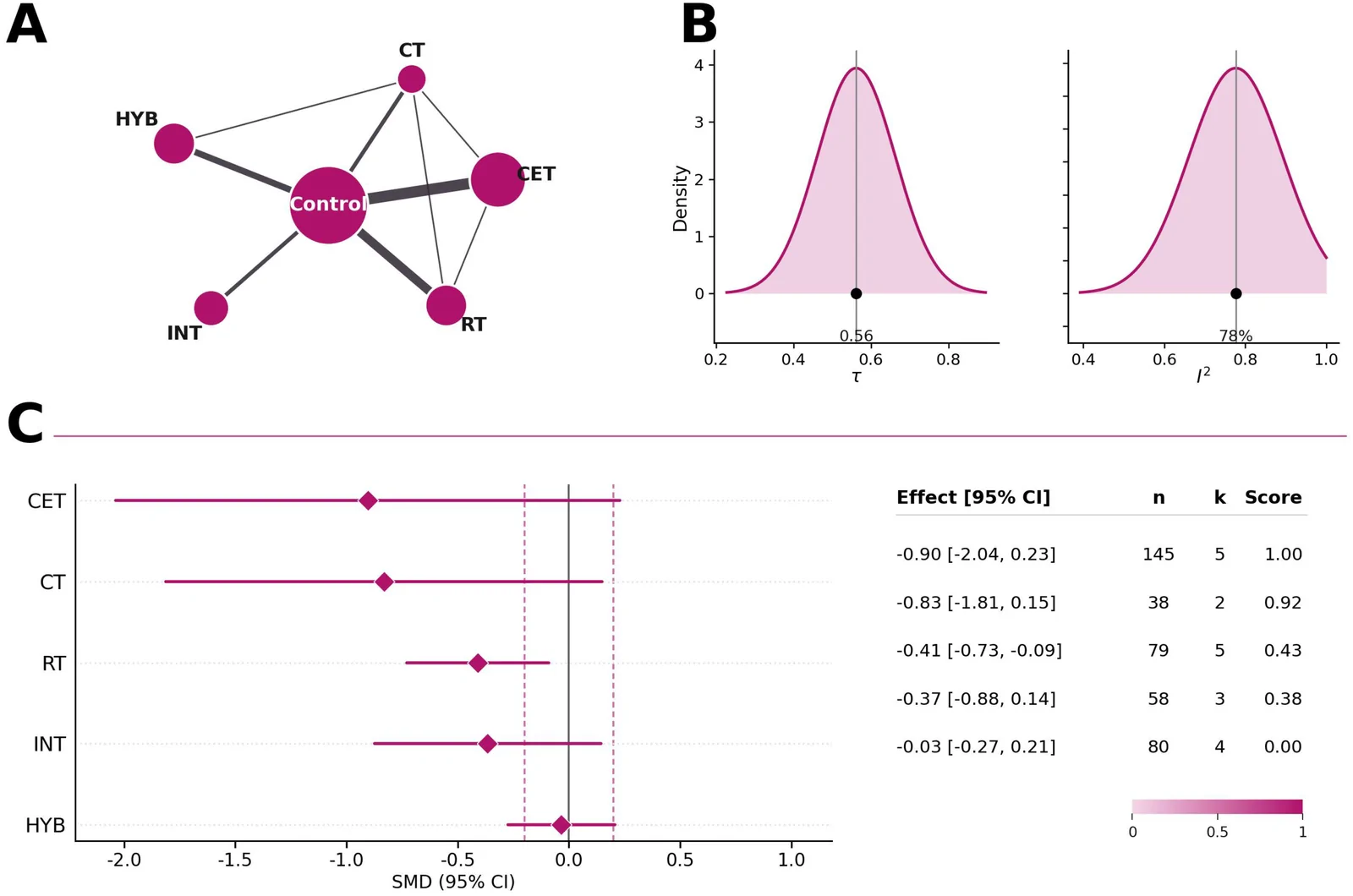
Network structure, heterogeneity distributions, and modality-specific effects for CIMT. **(A)** shows the evidence network, **(B)** shows heterogeneity distributions, and **(C)** shows treatment-versus-control estimates and rank scores.

Exercise was associated with improved FMD (SMD, 0.99; 95% CI, 0.69 to 1.29), reduced PWV (SMD, -0.31; 95% CI, -0.44 to -0.18), and reduced CIMT (SMD, -0.20; 95% CI, -0.36 to -0.05). **Figures 2**–**4** summarize the outcome-specific networks: panel A shows the evidence geometry, panel B shows heterogeneity distributions, and panel C shows modality-specific treatment-versus-control estimates with descriptive rank scores.

For FMD, the apparent effect was large but heterogeneity was substantial (I2 = 87.1%). HYB had the largest descriptive rank score, followed by CET and INT, but HYB evidence was sparse and imprecise; therefore, this ranking should be considered exploratory rather than definitive (**Figure 2**). For PWV, INT had the largest reduction, followed by RT and HYB (**Figure 3**). For CIMT, CET and CT had the largest estimated reductions, but both estimates had wide intervals, whereas RT showed a smaller but more precise reduction (**Figure 4**).

### Exploration of heterogeneity, small-study effects, and sensitivity analyses

Exploratory subgroup analyses by intervention frequency, duration, region, and sex are provided in **Supplementary Table 3** rather than in the main text. These analyses suggested that regional and sex-related patterns may exist, but the results are ecological and may reflect differences in trial design, supervision, baseline risk, adherence, or vascular measurement protocols rather than causal effect modification.

**Figure 5:**
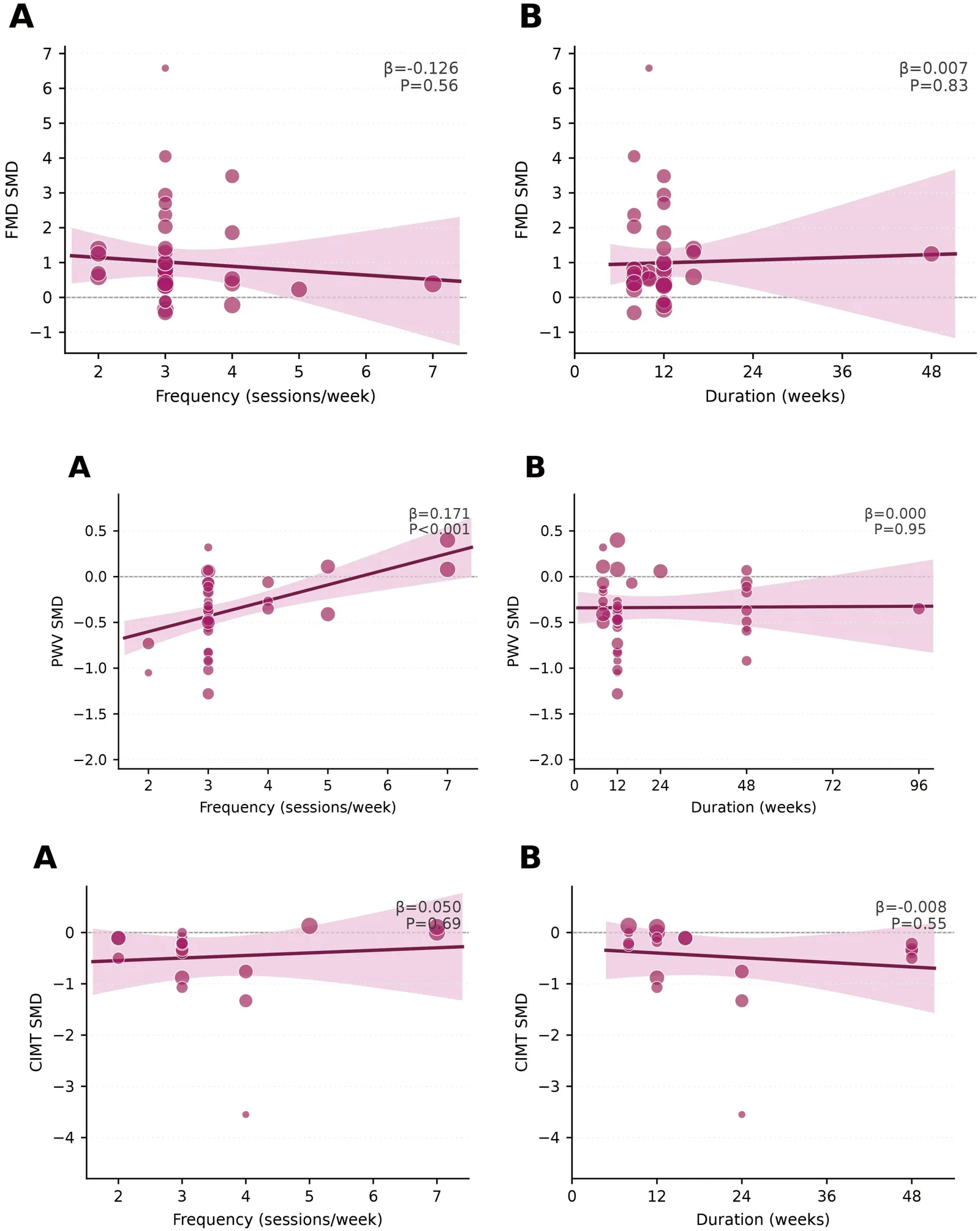
Meta-regression of intervention frequency and duration across FMD, PWV, and CIMT. Points are effect sizes; shaded areas represent 95% confidence bands. **(A)** frequency and **(B)** duration for each row (FMD upper row, PWV middle row, CIMT lower row).

Meta-regression of intervention frequency and duration is shown in **Figure 5**. Frequency was associated with PWV effects (beta = 0.171; P < 0.001), whereas frequency and duration were not clearly associated with FMD or CIMT effects, and duration was not clearly associated with PWV effects. These findings should be interpreted cautiously because meta-regression used aggregate trial-level data and was sensitive to sparse values at higher frequencies and longer durations.

**Figure 6:**
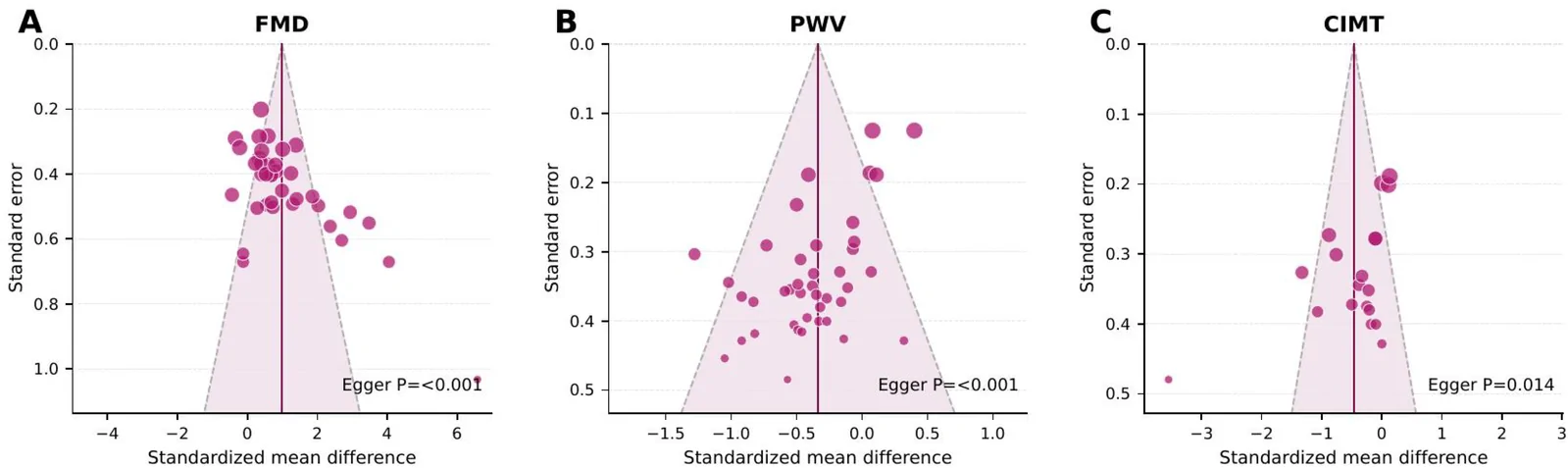
Funnel plots for small-study effects across FMD, PWV, and CIMT. **(A)** shows the funnel plot for FMD, **(B)** shows the funnel plot for PWV, and **(C)** shows the funnel plot for CIMT.

Funnel plots indicated possible small-study effects for all three outcomes (Egger tests: FMD, P < 0.001; PWV, P < 0.001; CIMT, P = 0.014), although asymmetry may also reflect true between-study heterogeneity, different exercise prescriptions, and outcome-measurement variability (**Figure 6**).

### Sensitivity and robustness analyses

Sensitivity and robustness analyses are summarized in **Supplementary Table 6**. Exclusion of studies judged at high risk of bias, exclusion of specialized cardiac or renal cohorts, and alternative assumptions for missing SDs of change scores did not materially change the direction of the findings for FMD, PWV, or CIMT. Some estimates became less precise after exclusions because evidence was sparse. Sensitivity plots were omitted because they were visually redundant with the primary analyses and did not alter the interpretation.

## Discussion

In this systematic review and Bayesian network meta-analysis of 51 randomized trials, exercise interventions were associated with favorable changes in endothelial function, arterial stiffness, and structural vascular remodeling among adults with overweight or obesity. The magnitude and consistency of effects differed by outcome. FMD showed the largest average improvement but also the greatest heterogeneity, whereas PWV and CIMT effects were smaller and more consistent. This pattern is biologically plausible because functional endothelial responses may occur earlier than changes in arterial stiffness or structural wall remodeling (Sun et al., 2024).

The comparative ranking patterns should be interpreted with caution. The descriptive rank scores shown in the outcome figures favored HYB for FMD, INT for PWV, and CET/CT for CIMT. However, confidence in these rankings is constrained by low CINeMA certainty, sparse data for several modalities, high heterogeneity for FMD, and imperfect transitivity across clinically and methodologically heterogeneous trials. Thus, the results identify plausible modality-specific patterns rather than definitive superiority of one modality over another.

The validity of indirect comparisons depends on the assumption that trials comparing different exercise modalities are sufficiently similar with respect to important effect modifiers. Although the common population definition, vascular outcome domains, and control conditions supported a connected evidence structure, intervention dose, comorbidity burden, region, supervision, adherence, and measurement protocols varied across nodes. Formal inconsistency tests did not reveal a major inconsistency signal where estimable, but the small number of closed loops limits the strength of this evidence. Consequently, the network estimates should be viewed as comparative summaries under plausible but imperfect transitivity rather than as definitive proof of superiority.

For FMD, several exercise modalities were associated with improvement. Continuous aerobic exercise and interval-based exercise may increase repeated shear-stress exposure, which can improve endothelial nitric oxide bioavailability and vasodilatory capacity (Green et al., 2017; Thijssen et al., 2019; Tao et al., 2023). The large estimate for HYB should be interpreted particularly carefully because HYB programs were heterogeneous and included mixed or technology-assisted interventions that may not share a uniform physiological mechanism. The high FMD heterogeneity also suggests that trial methods, cuff position, operator expertise, baseline cardiometabolic risk, and adherence may strongly influence estimated effects (Thijssen et al., 2019).

For PWV, reductions were generally modest but clinically directionally consistent. Interval training ranked highest in the descriptive analysis, while RT and HYB also showed favorable estimates. PWV reflects both structural arterial wall properties and functional vascular tone (Park et al., 2022). Exercise may reduce arterial stiffness through improved blood pressure regulation, autonomic balance, endothelial function, and cardiorespiratory fitness (Lopes et al., 2021). However, because some interval and hybrid comparisons were based on relatively few trials, modality-specific conclusions should remain provisional.

For CIMT, exercise produced a small average reduction, with larger descriptive estimates for CET and CT and a more precise estimate for RT. CIMT is a structural marker that usually changes slowly; therefore, small short-term effects are expected, and longer interventions may be required to detect consistent remodeling (Willeit et al., 2020; Wang et al., 2022). Aerobic and resistance training may affect CIMT through complementary hemodynamic, metabolic, inflammatory, and blood pressure pathways (Heffernan et al., 2013; Byrkjeland et al., 2016; Pae et al., 2023).

Regional and sex-related subgroup patterns were retained in the Supplementary Materials to avoid overemphasizing ecological comparisons in the main text. Such differences could reflect methodological variation, baseline risk, sex distribution, supervision, diet, background physical activity, or outcome measurement protocols rather than true biological effect modification. These results should therefore be used to generate hypotheses for future trials rather than to guide definitive subgroup-specific prescriptions.

### Strengths and limitations

This review has several strengths. It followed a registered protocol, used PRISMA 2020 and PRISMA-NMA guidance, included only randomized trials, compared multiple exercise modalities within a network framework, assessed risk of bias with RoB 2, evaluated evidence confidence using CINeMA, and synthesized three complementary vascular outcomes. We also explicitly assessed network assumptions, small-study effects, subgroup patterns, and sensitivity analyses.

Important limitations remain. First, FMD heterogeneity was substantial and was not fully explained by subgroup analysis or meta-regression. Second, transitivity was plausible but imperfect because intervention dose, comorbidity profile, supervision, adherence, and vascular measurement protocols varied across nodes, and inconsistency tests were underpowered in sparse networks. Third, classification of complex exercise protocols required judgment, especially for CT and HYB, although alternative classification robustness checks did not materially change the direction of findings. Fourth, change-score analyses were used to address baseline imbalance, but imputed SDs of change may affect precision. Fifth, some comparisons were sparse, making rank scores unstable. Sixth, small-study effects were suggested by funnel plots, although asymmetry may reflect heterogeneity rather than publication bias alone. Seventh, trials enrolling participants with cardiac, renal, diabetic, or hypertensive comorbidities were included only when the overweight/obesity criterion was met; this improves clinical relevance to obesity-associated multimorbidity but may reduce generalizability to otherwise healthy adults with uncomplicated obesity. Finally, overall CINeMA certainty was low, and modality rankings should not be treated as definitive clinical recommendations.

## Conclusion

Exercise interventions are associated with improvements in vascular health among adults with overweight or obesity. The available evidence suggests possible modality-specific patterns, with HYB ranking highest for FMD, INT for PWV, and CET/CT for CIMT in descriptive treatment-versus-control analyses. However, because the certainty of evidence was low, FMD heterogeneity was high, indirect comparisons relied on plausible but imperfect transitivity, and several comparisons were sparse, these rankings should be interpreted as hypothesis-generating. Exercise prescriptions should be individualized according to the target vascular outcome, comorbidities, safety, feasibility, patient preference, and long-term adherence.

## Data Availability

The raw data supporting the conclusions of this article will be made available by the authors, without undue reservation.
