# Supplementary material for: Which exercise modality improves vascular function most in overweight or obese adults? A Bayesian network meta-analysis of randomized controlled trials
Source: Front Physiol. 2026 Aug 7;17:1806291. doi: 10.3389/fphys.2026.1806291 (PMC13494261; doi:10.3389/fphys.2026.1806291)
Supplement: Supplementary file 1 [file Table1.docx]

**Supplementary Materials**

Comparative Effects of Exercise Modalities on Vascular Function in Adults With Overweight or Obesity

# **Table S1. Search strategy**

| #1 | overweight OR obese OR "body mass index" OR BMI OR "body size" OR "fat mass" OR adult OR adults |
| --- | --- |
| #2 | exercise OR "aerobic exercise" OR "resistance training" OR "interval training" OR "strength training" OR "combined training" OR "hybrid training" |
| #3 | atherosclerosis OR "arterial stiffness" OR "carotid atherosclerosis" OR "cardiovascular health" |
| #4 | "flow mediated dilation" OR FMD |
| #5 | "pulse wave velocity" OR PWV |
| #6 | "intima media thickness" OR IMT |
| #7 | "endothelial function" OR "vascular function" |
| #8 | "arterial compliance" OR "arterial elasticity" |
| #9 | "arterial remodeling" OR "vascular remodeling" |
| #10 | "subclinical atherosclerosis" OR "preclinical atherosclerosis" |
| E1 | #1 AND #2 AND #3 AND #4 |
| E2 | #1 AND #2 AND #3 AND #5 |
| E3 | #1 AND #2 AND #3 AND #6 |
| E4 | #1 AND #2 AND #3 AND #7 |
| E5 | #1 AND #2 AND #3 AND #8 |
| E6 | #1 AND #2 AND #3 AND #9 |
| E7 | #1 AND #2 AND #3 AND #10 |

| **Database** | **strategy** | **Search strategy details** |
| --- | --- | --- |
|  | E1 | (overweight OR obese OR BMI OR adult) AND (exercise OR resistance training OR aerobic OR interval) AND (atherosclerosis OR arterial stiffness) AND (flow mediated dilation OR FMD) |
|  | E2 | (overweight OR obese OR BMI OR adult) AND (exercise OR resistance training OR aerobic OR interval) AND (arterial stiffness) AND (pulse wave velocity OR PWV) |
|  | E3 | (overweight OR obese OR BMI OR adult) AND (exercise OR resistance training OR aerobic OR interval) AND (atherosclerosis) AND (intima media thickness OR IMT) |
| PubMed | E4 | (overweight OR obese OR BMI OR adult) AND (exercise OR resistance training OR aerobic OR interval) AND (blood pressure OR hypertension OR SBP OR DBP) |
|  | E5 | (overweight OR obese OR BMI OR adult) AND (exercise OR resistance training OR aerobic OR interval) AND (lipids OR cholesterol OR triglycerides OR LDL OR HDL) |
|  | E6 | (overweight OR obese OR BMI OR adult) AND (exercise OR resistance training OR aerobic OR interval) AND (glucose OR insulin OR HbA1c OR HOMA-IR) |
|  | E7 | (overweight OR obese OR BMI OR adult) AND (exercise OR resistance training OR aerobic OR interval) AND (inflammation OR CRP OR hsCRP OR IL-6 OR TNF-α) |
|  | E1 | (overweight OR obese OR BMI OR adult) AND (exercise OR resistance training OR aerobic OR interval) AND (atherosclerosis OR arterial stiffness) AND (flow mediated dilation OR FMD) |
|  | E2 | (overweight OR obese OR BMI OR adult) AND (exercise OR resistance training OR aerobic OR interval) AND (arterial stiffness) AND (pulse wave velocity OR PWV) |
|  | E3 | (overweight OR obese OR BMI OR adult) AND (exercise OR resistance training OR aerobic OR interval) AND (atherosclerosis) AND (intima media thickness OR IMT) |
| Web of Science | E4 | (overweight OR obese OR BMI OR adult) AND (exercise OR resistance training OR aerobic OR interval) AND (blood pressure OR hypertension OR SBP OR DBP) |
|  | E5 | (overweight OR obese OR BMI OR adult) AND (exercise OR resistance training OR aerobic OR interval) AND (lipids OR cholesterol OR triglycerides OR LDL OR HDL) |
|  | E6 | (overweight OR obese OR BMI OR adult) AND (exercise OR resistance training OR aerobic OR interval) AND (glucose OR insulin OR HbA1c OR HOMA-IR) |
|  | E7 | (overweight OR obese OR BMI OR adult) AND (exercise OR resistance training OR aerobic OR interval) AND (inflammation OR CRP OR hsCRP OR IL-6 OR TNF-α) |
|  | E1 | (overweight OR obese OR BMI OR adult) AND (exercise OR resistance training OR aerobic OR interval) AND (atherosclerosis OR arterial stiffness) AND (flow mediated dilation OR FMD) |
|  | E2 | (overweight OR obese OR BMI OR adult) AND (exercise OR resistance training OR aerobic OR interval) AND (arterial stiffness) AND (pulse wave velocity OR PWV) |
|  | E3 | (overweight OR obese OR BMI OR adult) AND (exercise OR resistance training OR aerobic OR interval) AND (atherosclerosis) AND (intima media thickness OR IMT) |
| SPORTDiscus | E4 | (overweight OR obese OR BMI OR adult) AND (exercise OR resistance training OR aerobic OR interval) AND (blood pressure OR hypertension OR SBP OR DBP) |
|  | E5 | (overweight OR obese OR BMI OR adult) AND (exercise OR resistance training OR aerobic OR interval) AND (lipids OR cholesterol OR triglycerides OR LDL OR HDL) |
|  | E6 | (overweight OR obese OR BMI OR adult) AND (exercise OR resistance training OR aerobic OR interval) AND (glucose OR insulin OR HbA1c OR HOMA-IR) |
|  | E7 | (overweight OR obese OR BMI OR adult) AND (exercise OR resistance training OR aerobic OR interval) AND (inflammation OR CRP OR hsCRP OR IL-6 OR TNF-α) |
|  | E1 | (overweight OR obese OR BMI OR adult) AND (exercise OR resistance training OR aerobic OR interval) AND (atherosclerosis OR arterial stiffness) AND (flow mediated dilation OR FMD) |
|  | E2 | (overweight OR obese OR BMI OR adult) AND (exercise OR resistance training OR aerobic OR interval) AND (arterial stiffness) AND (pulse wave velocity OR PWV) |
|  | E3 | (overweight OR obese OR BMI OR adult) AND (exercise OR resistance training OR aerobic OR interval) AND (atherosclerosis) AND (intima media thickness OR IMT) |
| Cochrane | E4 | (overweight OR obese OR BMI OR adult) AND (exercise OR resistance training OR aerobic OR interval) AND (blood pressure OR hypertension OR SBP OR DBP) |
|  | E5 | (overweight OR obese OR BMI OR adult) AND (exercise OR resistance training OR aerobic OR interval) AND (lipids OR cholesterol OR triglycerides OR LDL OR HDL) |
|  | E6 | (overweight OR obese OR BMI OR adult) AND (exercise OR resistance training OR aerobic OR interval) AND (glucose OR insulin OR HbA1c OR HOMA-IR) |
|  | E7 | (overweight OR obese OR BMI OR adult) AND (exercise OR resistance training OR aerobic OR interval) AND (inflammation OR CRP OR hsCRP OR IL-6 OR TNF-α) |

Core database strategy: (#1 AND #2) combined with vascular outcome concepts (#3 to #10). The search was adapted for PubMed, Web of Science, the Cochrane Library, and EBSCO SPORTDiscus, with database-specific syntax applied as required.

# **Table S2. Characteristics of included randomized controlled trials**

References in this table correspond to the supplementary reference list (S1-S51).

| **Study** | **Countries** | **RCT arms** | **n (intervention)** | **n (control)** | **Intervention characteristics** | **Intervention characteristics** | **Intervention characteristics** | **Main outcomes** | **Adverse effects** |
| --- | --- | --- | --- | --- | --- | --- | --- | --- | --- |
| Aispuru-Lanche et al.(2024)[S1] | Spain | 3 | 28 | 24 | INT | 16 | 2 | FMD、CIMT | Not reported |
|  |  |  | 28 | 24 | INT |  |  |  |  |
| Banks et al.(2024)[S2] | United States | 2 | 13 | 13 | RT | 9 | 3 | FMD、PWV | Not reported |
| Barone Gibbs et al.(2024)[S3] | United State | 2 | 134 | 129 | HYB | 12 | 7 | PWV | No adverse events |
| Cox et al.(2024)[S4] | Australia | 3 | 11 | 15 | HYB | 8 | 3 | FMD、PWV | No adverse events |
|  |  |  | 15 | 15 | CT |  | 4 |  |  |
| Taha et al.(2023)[S5] | Saudi Arabia | 2 | 25 | 28 | INT | 12 | 3 | PWV | Not reported |
| Twerenbold et al.(2023)[S6] | Switzerland | 2 | 19 | 19 | INT | 8 | 3 | FMD | Not reported |
| He et al.(2022)[S7] | China | 4 | 15 | 15 | CET | 8 | 5 | FMD | No adverse events |
|  |  |  | 8 | 15 | CET | 8 | 3 |  |  |
|  |  |  | 10 | 15 | INT | 8 | 3 |  |  |
| Hovsepian et al.(2021)[S8] | Iran | 2 | 14 | 12 | INT | 10 | 4 | FMD | One member's asthma triggered by HIIT |
| Turri-Silva et al.(2021)[S9] | Brazil | 3 | 5 | 4 | INT | 12 | 3 | FMD | Not reported |
|  |  |  | 6 | 4 | RT |  |  |  |  |
| Cahu Rodrigues et al.(2020)[S10] | Brazil | 2 | 17 | 16 | RT | 12 | 3 | FMD、PWV | No adverse events |
| Claes et al.(2020)[S11] | Belgium | 2 | 60 | 60 | HYB | 12 | 7 | FMD、CIMT | 4 dropouts due to SAEs |
| Ghardashi-Afousi et al.(2020)[S12] | Iran | 2 | 30 | 29 | INT | 12 | 3 | CIMT | Not reported |
| Gholami et al.(2020)[S13] | Iran | 2 | 16 | 15 | CET | 12 | 3 | FMD、CIMT | No adverse events |
| Hetherington-Rauth et al.(2020)[S14] | Portugal | 2 | 14 | 22 | HYB | 48 | 3 | PWV、CIMT | Not reported |
| Ho et al.(2020)[S15] | Australia | 2 | 30 | 30 | INT | 8 | 3 | PWV | No adverse events |
| Jo et al.(2020)[S16] | South Korea | 3 | 21 | 13 | HYB | 12 | 4 | FMD | No adverse events |
|  |  |  | 13 | 13 | CET |  |  |  |  |
| Jones et al.(2020)[S17] | New Zealand | 2 | 26 | 25 | CT | 12 | 2 | PWV | No adverse events |
| Wong et al.(2020)[S18] | United States | 2 | 14 | 14 | RT | 12 | 3 | PWV | Not reported |
| Kirkman et al.(2019)[S19] | United States | 2 | 15 | 16 | CET | 12 | 3 | PWV、FMD | No adverse events |
| Magalhães et al.(2019)[S20] | Portugal | 3 | 16 | 22 | CT | 48 | 3 | PWV、CIMT | Not reported |
|  |  |  | 13 | 22 | HYB |  |  |  |  |
| Rahbar et al.(2018)[S21] | Iran | 2 | 13 | 15 | CET | 8 | 3 | CIMT | Not reported |
| Slivovskaja et al.(2018)[S22] | Lithuania | 2 | 84 | 42 | CET | 8 | 5 | PWV、CIMT | Not reported |
| Azadpour et al.(2017)[S23] | Istanbul | 2 | 12 | 12 | CET | 10 | 3 | FMD | Not reported |
| Bellia et al.(2017)[S24] | Italy | 2 | 11 | 11 | INT | 12 | 2 | PWV | Not reported |
| DeVallance et al.(2016)[S25] | United States | 2 | 13 | 16 | RT | 8 | 3 | CIMT、PWV | Not reported |
| Robinson et al.(2016)[S26] | United States | 2 | 10 | 9 | CET | 8 | 3 | FMD | Not reported |
| Almenning et al.(2015)[S27] | Norway | 3 | 8 | 9 | INT | 10 | 3 | FMD | Not reported |
|  |  |  | 8 | 9 | RT |  |  |  |  |
| Figueroa(1) et al.(2015)[S28] | United States | 2 | 12 | 12 | HYB | 12 | 3 | PWV | Not reported |
| Figueroa(2) et al.(2014)[S29] | United States | 2 | 13 | 12 | HYB | 12 | 3 | PWV | Not reported |
| Franklin et al.(2015)[S30] | United States | 2 | 10 | 8 | RT | 8 | 2 | FMD | Not reported |
| Greenwood(1) et al.(2015)[S31] | United Kingdom | 3 | 13 | 20 | CET | 12 | 3 | PWV | Not reported |
|  |  |  |  |  | RT |  |  |  |  |
| Greenwood(2) et al.(2015)[S32] | United Kingdom | 2 | 8 | 10 | CT | 48 | 3 | PWV | Not reported |
| Oliveira et al.(2015)[S33] | Portugal | 2 | 37 | 41 | CET | 8 | 3 | PWV | Not reported |
| Van Craenenbroeck et al.(2015)[S34] | Belgium | 2 | 19 | 21 | CET | 12 | 4 | FMD | Not reported |
| Croymans et al.(2014)[S35] | United States | 2 | 28 | 8 | RT | 12 | 3 | PWV、CIMT | Not reported |
| Donley et al.(2014)[S36] | United States | 2 | 11 | 11 | CET | 8 | 3 | PWV、CIMT | Not reported |
| Headley et al.(2014)[S37] | United States | 2 | 25 | 21 | CET | 16 | 3 | PWV | Not reported |
| Pugh et al.(2014)[S38] | United Kingdom | 2 | 13 | 8 | CET | 16 | 3 | FMD | Not reported |
| Beck et al.(2013)[S39] | United States | 3 | 15 | 15 | RT | 8 | 3 | FMD | Not reported |
|  |  |  | 13 | 15 | CET |  |  |  |  |
| Heydari et al.(2013)[S40] | Australia | 2 | 20 | 18 | INT | 12 | 3 | PWV | Not reported |
| Kadoglou et al.(2013)[S41] | Greece | 4 | 23 | 24 | RT | 24 | 4 | CIMT | Not reported |
|  |  |  | 21 | 24 | CET |  |  |  |  |
|  |  |  | 22 | 24 | CT |  |  |  |  |
| Dobrosielski et al.(2012)[S42] | United States | 2 | 51 | 63 | CT | 24 | 3 | PWV | Not reported |
| Molmen-Hansen et al.(2012)[S43] | Norway | 3 | 25 | 25 | INT | 12 | 3 | FMD | Not reported |
|  |  |  | 23 | 25 | CET |  |  |  |  |
| Nualnim et al.(2012)[S44] | United States | 2 | 24 | 19 | CET | 12 | 3 | PWV、FMD | Not reported |
| Hermann et al.(2011)[S45] | Denmark | 2 | 14 | 13 | INT | 8 | 3 | FMD | Not reported |
| Stensvold et al.(2010)[S46] | Norway | 4 | 11 | 11 | INT | 12 | 3 | FMD | Not reported |
|  |  |  | 11 | 11 | RT |  |  |  |  |
|  |  |  | 10 | 11 | HYB |  |  |  |  |
| Loimaala(1) et al.(2009)[S47] | Finland | 2 | 24 | 24 | CT | 96 | 4 | PWV | Not reported |
| Loimaala(2) et al.(2003)[S48] | Finland | 2 | 24 | 25 | CT | 48 | 4 | PWV | Not reported |
| Braith et al.(2008)[S49] | United States | 2 | 9 | 7 | CET | 12 | 3 | FMD | Not reported |
| Bircher et al.(2007)[S50] | Germany | 2 | 13 | 13 | CET | 12 | 3 | FMD | Not reported |
| Olson et al.(2006)[S51] | United States | 2 | 15 | 15 | RT | 48 | 2 | FMD、CIMT | Not reported |

**Figure S1. Risk-of-bias assessment by trial**

Figure S1. RoB 2 traffic-light plot for randomized controlled trials.

# **Figure S2. Summary risk-of-bias assessment**

Figure S2. RoB 2 domain-level summary.

# **Table S3. Complete subgroup analysis**

| Outcome | Factor | Subgroup | k | n | % | SMD (95% CI) | P value | P for interaction |
| --- | --- | --- | --- | --- | --- | --- | --- | --- |
| FMD | Frequency | <3 sessions/week | 4 | 156 | 13.9 | 0.86 [0.47, 1.25] | <0.001 | 0.640 |
| FMD | Frequency | 3 sessions/week | 26 | 766 | 68.3 | 0.94 [0.58, 1.29] | <0.001 | 0.640 |
| FMD | Frequency | >3 sessions/week | 7 | 199 | 17.8 | 1.35 [0.41, 2.30] | 0.005 | 0.640 |
| FMD | Duration | <12 weeks | 16 | 404 | 36.1 | 1.08 [0.57, 1.58] | <0.001 | 0.832 |
| FMD | Duration | 12 weeks | 17 | 562 | 50.1 | 0.91 [0.46, 1.36] | <0.001 | 0.832 |
| FMD | Duration | >12 weeks | 4 | 155 | 13.8 | 1.08 [0.67, 1.50] | <0.001 | 0.832 |
| PWV | Frequency | <3 sessions/week | 2 | 73 | 3.4 | -0.82 [-1.30, -0.34] | 0.001 | <0.001 |
| PWV | Frequency | 3 sessions/week | 32 | 1179 | 54.7 | -0.36 [-0.48, -0.24] | <0.001 | <0.001 |
| PWV | Frequency | >3 sessions/week | 7 | 905 | 41.9 | 0.07 [-0.06, 0.21] | 0.275 | <0.001 |
| PWV | Duration | <12 weeks | 10 | 545 | 25.3 | -0.20 [-0.37, -0.03] | 0.024 | 0.942 |
| PWV | Duration | 12 weeks | 19 | 1082 | 50.2 | -0.18 [-0.30, -0.06] | 0.004 | 0.942 |
| PWV | Duration | >12 weeks | 12 | 530 | 24.5 | -0.21 [-0.39, -0.04] | 0.016 | 0.942 |
| CIMT | Frequency | <3 sessions/week | 3 | 134 | 14.2 | -0.19 [-0.53, 0.15] | 0.268 | 0.026 |
| CIMT | Frequency | 3 sessions/week | 10 | 350 | 36.9 | -0.41 [-0.64, -0.19] | <0.001 | 0.026 |
| CIMT | Frequency | >3 sessions/week | 6 | 464 | 48.9 | -0.01 [-0.20, 0.17] | 0.885 | 0.026 |
| CIMT | Duration | <12 weeks | 4 | 205 | 21.6 | 0.01 [-0.27, 0.30] | 0.922 | 0.317 |
| CIMT | Duration | 12 weeks | 6 | 362 | 38.2 | -0.22 [-0.44, -0.00] | 0.045 | 0.317 |
| CIMT | Duration | >12 weeks | 9 | 381 | 40.2 | -0.24 [-0.45, -0.04] | 0.019 | 0.317 |
| FMD | Region | Asia | 8 | 219 | 19.5 | 2.34 [1.28, 3.39] | <0.001 | 0.009 |
| FMD | Region | Europe | 15 | 553 | 49.3 | 0.87 [0.46, 1.28] | <0.001 | 0.009 |
| FMD | Region | America | 10 | 235 | 21.0 | 0.52 [0.20, 0.85] | 0.002 | 0.009 |
| FMD | Region | Oceania | 4 | 114 | 10.2 | 0.55 [0.19, 0.92] | 0.003 | 0.009 |
| FMD | Sex | Male | 1 | 31 | 2.8 | NA | NA | <0.001 |
| FMD | Sex | Female | 11 | 270 | 24.1 | 1.72 [0.78, 2.66] | <0.001 | <0.001 |
| FMD | Sex | Mixed | 25 | 820 | 73.1 | 0.67 [0.37, 0.97] | <0.001 | <0.001 |
| PWV | Region | Asia | 1 | 53 | 2.5 | -1.28 [-1.87, -0.68] | <0.001 | <0.001 |
| PWV | Region | Europe | 17 | 824 | 38.2 | -0.34 [-0.48, -0.19] | <0.001 | <0.001 |
| PWV | Region | America | 19 | 1113 | 51.6 | -0.03 [-0.15, 0.09] | 0.658 | <0.001 |
| PWV | Region | Oceania | 4 | 167 | 7.7 | -0.34 [-0.65, -0.03] | 0.029 | <0.001 |
| PWV | Sex | Male | 4 | 171 | 7.9 | -0.26 [-0.57, 0.05] | 0.102 | <0.001 |
| PWV | Sex | Female | 9 | 314 | 14.6 | -0.58 [-0.81, -0.36] | <0.001 | <0.001 |
| PWV | Sex | Mixed | 28 | 1672 | 77.5 | -0.11 [-0.21, -0.02] | 0.024 | <0.001 |
| CIMT | Region | Asia | 3 | 118 | 12.4 | -0.76 [-1.13, -0.38] | <0.001 | 0.004 |
| CIMT | Region | Europe | 13 | 749 | 79.0 | -0.08 [-0.23, 0.07] | 0.289 | 0.004 |
| CIMT | Region | America | 3 | 81 | 8.6 | -0.27 [-0.71, 0.17] | 0.224 | 0.004 |
| CIMT | Sex | Male | 3 | 103 | 10.9 | -0.47 [-0.92, -0.02] | 0.040 | 0.264 |
| CIMT | Sex | Female | 1 | 30 | 3.2 | NA | NA | 0.264 |
| CIMT | Sex | Mixed | 15 | 815 | 85.9 | -0.14 [-0.28, 0.00] | 0.050 | 0.264 |

Abbreviations: k, number of effect sizes; n, number of participants. Percentages refer to the proportion of participants contributing to the corresponding outcome-specific subgroup analysis.

**Supplementary assessment of network assumptions**

Assessment of transitivity and network consistency. To address the assumptions of network meta-analysis, we compared key potential effect modifiers across intervention nodes and assessed consistency where the network geometry permitted. The evidence networks were largely anchored by control comparisons and had few independent active-active closed loops; therefore, inconsistency findings were interpreted cautiously.

**Table S4. Assessment of transitivity across intervention nodes**

| Node | Evidence base | Duration and frequency | Clinical/methodological comparability | Implication for transitivity |
| --- | --- | --- | --- | --- |
| CET | 21 intervention arms; 407 participants in exercise arms. Outcomes included FMD, PWV, and CIMT. | Median duration 12 weeks (range 8-24); median frequency 3 sessions/week (range 3-5). | Broadest evidence base and multiple regions; protocols were mostly continuous aerobic/endurance exercise. Some trials enrolled comorbid cardiometabolic, renal, or cardiovascular populations. | Generally comparable to other exercise nodes for target population and control type, but differences in comorbidity and measurement protocols may contribute to heterogeneity. |
| RT | 13 intervention arms; 173 participants in exercise arms. Outcomes included FMD, PWV, and CIMT. | Median duration 12 weeks (range 8-48); median frequency 3 sessions/week (range 2-4). | Resistance prescriptions varied in load, circuit structure, and supervision. Evidence was smaller than CET and included several small trials. | Transitivity considered plausible but uncertain because resistance dose and participant risk profiles varied. |
| INT | 15 intervention arms; 278 participants in exercise arms. Outcomes mainly FMD and PWV, with fewer CIMT data. | Median duration 12 weeks (range 8-16); median frequency 3 sessions/week (range 2-4). | Protocols included HIIT, sprint interval, and aerobic interval training; intensity definitions and recovery periods differed across trials. | Indirect comparisons involving INT should be interpreted cautiously because training intensity and interval structure were heterogeneous. |
| CT | 8 intervention arms; 186 participants in exercise arms. Outcomes mainly PWV and CIMT, with fewer FMD data. | Median duration 36 weeks (range 8-96); median frequency 3.5 sessions/week (range 2-4). | CT trials often had longer duration than other nodes and combined separately prescribed aerobic/endurance and resistance components. | Duration imbalance may influence indirect comparisons, especially for structural outcomes such as CIMT. |
| HYB | 9 intervention arms; 288 participants in exercise arms. Outcomes included FMD, PWV, and CIMT. | Median duration 12 weeks (range 8-48); median frequency 3 sessions/week (range 3-7). | Most heterogeneous node: mixed, recreational, technology-assisted, vibration, sedentary-behavior-reduction, or rehabilitation-platform interventions. | Transitivity is most uncertain for HYB because protocols may differ substantially in physiological stimulus and non-exercise co-intervention components. |
| Overall judgement | All active nodes were compared with usual lifestyle, no-exercise control, conventional therapy, or standard care. | Most interventions lasted 8-12 weeks, but CT and some HYB/RT trials were longer. | The target population was adults with overweight or obesity, but comorbidity profiles, supervision, adherence, and outcome-assessment protocols varied. | Transitivity was considered plausible for broad exercise-versus-control inference, but only partially satisfied for fine-grained modality rankings; rankings were therefore treated as exploratory. |

Abbreviations: CET, continuous endurance training; CT, combined training; FMD, flow-mediated dilation; HYB, hybrid/mixed-modality programs; INT, interval training; PWV, pulse wave velocity; RT, resistance training; CIMT, carotid intima-media thickness.

**Table S5. Network consistency and inconsistency assessment**

| Outcome | Network geometry | Consistency/inconsistency assessment | Interpretation |
| --- | --- | --- | --- |
| FMD | Connected network including CET, RT, INT, CT, HYB, and control. Active-active information was limited and was mostly generated by multi-arm trials. | Closed loops were sparse and supported by few independent comparisons. Formal node-splitting/design-by-treatment testing was therefore not stable for most active-active contrasts. Where direct and indirect evidence could be compared, no major qualitative inconsistency signal was observed. | Indirect estimates and rank scores should be interpreted as descriptive. High FMD heterogeneity remains a more important limitation than detectable inconsistency. |
| PWV | Control-anchored network with direct comparisons for all active nodes and limited active-active links, mainly from multi-arm trials. | Formal inconsistency assessment was limited by the small number of independent closed loops. No clear discordance between direct and indirect evidence was evident in assessable comparisons. | Exercise-versus-control inference was considered more robust than active-active rankings; modality rankings remain exploratory. |
| CIMT | Sparse network with fewer effect sizes and limited active-active evidence. | Closed-loop evidence was insufficient for robust statistical inconsistency testing across most contrasts. No obvious qualitative inconsistency was detected, but power to detect inconsistency was low. | Consistency could not be firmly established; CIMT rankings should be viewed as low-certainty and hypothesis-generating. |
| Overall judgement | Networks were connected but primarily star-shaped around the control node. | Absence of a strong inconsistency signal should not be interpreted as proof of consistency because independent closed loops were limited. | The validity of indirect comparisons depends on the partial transitivity judgement above; conclusions were tempered accordingly in the main manuscript. |

Because several networks contained few independent active-active closed loops, inconsistency testing had low power. These assessments were therefore used to support cautious interpretation rather than to prove the validity of all indirect comparisons.

# **Table S6. Sensitivity and robustness interpretation**

| **Analysis** | **Purpose** | **Result** | **Interpretation** |
| --- | --- | --- | --- |
| Exclusion of studies judged at high risk of bias | Assess whether high-risk trials drove pooled effects or rankings | The direction of the pooled effects remained consistent for FMD, PWV, and CIMT; precision decreased in sparse comparisons. | No material change in interpretation; sensitivity plots omitted because results were visually redundant with primary analyses. |
| Exclusion of specialized cardiac or renal cohorts | Assess whether cardiac rehabilitation, transplant, or chronic kidney disease cohorts drove the findings | The direction of effect remained favorable for exercise across vascular domains; no clear reversal of modality patterns was observed. | Findings are robust in direction but still limited by low certainty and reduced precision after exclusions. |
| Comparison of change-score assumptions | Assess the influence of missing SDs for change scores | Alternative assumptions did not produce an obvious change in the direction of results. | Magnitude estimates should be interpreted cautiously; direction of benefit was stable. |

# **Supplementary references**

S1. Aispuru-Lanche, R.; Jayo-Montoya, J.A.; Maldonado-Martín, S. Vascular-Endothelial Adaptations Following Low and High Volumes of High-Intensity Interval Training in Patients after Myocardial Infarction. Ther. Adv. Cardiovasc. Dis. 2024, 18, 17539447241286036, doi:10.1177/17539447241286036.

S2. Banks, N.F.; Rogers, E.M.; Stanhewicz, A.E.; Whitaker, K.M.; Jenkins, N.D.M. Resistance Exercise Lowers Blood Pressure and Improves Vascular Endothelial Function in Individuals with Elevated Blood Pressure or Stage-1 Hypertension. Am. J. Physiol. Heart Circ. Physiol. 2024, 326, H256–H269, doi:10.1152/ajpheart.00386.2023.

S3. Barone Gibbs, B.; Perera, S.; Huber, K.A.; Paley, J.L.; Conroy, M.B.; Jakicic, J.M.; Muldoon, M.F. Effects of Sedentary Behavior Reduction on Blood Pressure in Desk Workers: Results from the RESET-BP Randomized Clinical Trial. Circulation 2024, 150, 1416–1427, doi:10.1161/CIRCULATIONAHA.123.068564.

S4. Cox, E.R.; Gajanand, T.; Keating, S.E.; Hordern, M.D.; Burton, N.W.; Green, D.J.; Ramos, J.S.; Ramos, M.V.; Fassett, R.G.; Cox, S.V.; et al. Effect of Low-Volume Combined Aerobic and Resistance High-Intensity Interval Training on Vascular Health in People with Type 2 Diabetes: A Randomised Controlled Trial. Eur J Appl Physiol 2024, 124, 2819–2833, doi:10.1007/s00421-024-05473-8.

S5. Taha, M.M.; Aneis, Y.M.; Hasanin, M.E.; Felaya, E.E.; Aldhahi, M.I.; Abdeen, H.A.A. Effect of High Intensity Interval Training on Arterial Stiffness in Obese Hypertensive Women: A Randomized Controlled Trial. Eur. Rev. Med. Pharmacol. Sci. 2023, 27, 4069-4079, doi:10.26355/eurrev_202305_32314.

S6. Twerenbold, S.; Hauser, C.; Gander, J.; Carrard, J.; Gugleta, K.; Hinrichs, T.; Schmidt‐Trucksäss, A.; Hanssen, H.; Streese, L. Short‐term High‐intensity Interval Training Improves Micro‐ but Not Macrovascular Function in Hypertensive Patients. Scand. J. Med. Sci. Sports 2023, 33, 1231–1241, doi:10.1111/sms.14343.

S7. He, H.; Wang, C.; Chen, X.; Sun, X.; Wang, Y.; Yang, J.; Wang, F. The Effects of HIIT Compared to MICT on Endothelial Function and Hemodynamics in Postmenopausal Females. J. Sci. Med. Sport 2022, 25, 364–371, doi:10.1016/j.jsams.2022.01.007.

S8. Hovsepian, V.; Marandi, S.; Esfarjani, F.; Zavar, R.; Sadeghi, M. The Effect of All Extremity High Intensity Interval Training on Athero-Protective Factors and Endothelial Function in Overweight and Obese Women. Int. J. Prev. Med. 2021, 12, 141, doi:10.4103/ijpvm.IJPVM_248_19.

S9. Turri-Silva, N.; Vale-Lira, A.; Verboven, K.; Quaglioti Durigan, J.L.; Hansen, D.; Cipriano, G. High-Intensity Interval Training versus Progressive High-Intensity Circuit Resistance Training on Endothelial Function and Cardiorespiratory Fitness in Heart Failure: A Preliminary Randomized Controlled Trial. PLOS One 2021, 16, e0257607, doi:10.1371/journal.pone.0257607.

S10. Cahu Rodrigues, S.L.; Farah, B.Q.; Silva, G.; Correia, M.; Pedrosa, R.; Vianna, L.; Ritti-Dias, R.M. Vascular Effects of Isometric Handgrip Training in Hypertensives. Clin. Exp. Hypertens. 2020, 42, 24–30, doi:10.1080/10641963.2018.1557683.

S11. Claes, J.; Cornelissen, V.; McDermott, C.; Moyna, N.; Pattyn, N.; Cornelis, N.; Gallagher, A.; McCormack, C.; Newton, H.; Gillain, A.; et al. Feasibility, Acceptability, and Clinical Effectiveness of a Technology-Enabled Cardiac Rehabilitation Platform (Physical Activity Toward Health-I): Randomized Controlled Trial. J. Med. Internet Res. 2020, 22, e14221, doi:10.2196/14221.

S12. Ghardashi-Afousi, A.; Davoodi, M.; Hesamabadi, B.K.-; Asvadi-Fard, M.; Bigi, M.A.B.; Izadi, M.R.; Gaeini, A.A. Improved Carotid Intima-Media Thickness-Induced High-Intensity Interval Training Associated with Decreased Serum Levels of Dkk-1 and Sclerostin in Type 2 Diabetes. J. Diabetes Complications 2020, 34, 107469, doi:10.1016/j.jdiacomp.2019.107469.

S13. Gholami, F.; Nazari, H.; Alimi, M. Cycle Training Improves Vascular Function and Neuropathic Symptoms in Patients with Type 2 Diabetes and Peripheral Neuropathy: A Randomized Controlled Trial. Exp. Gerontol. 2020, 131, 110799, doi:10.1016/j.exger.2019.110799.

S14. Hetherington-Rauth, M.; Magalhães, J.P.; Júdice, P.B.; Melo, X.; Sardinha, L.B. Vascular Improvements in Individuals with Type 2 Diabetes Following a 1 Year Randomised Controlled Exercise Intervention, Irrespective of Changes in Cardiorespiratory Fitness. Diabetologia 2020, 63, 722–732, doi:10.1007/s00125-020-05089-5.

S15. Ho, T.Y.; Redmayne, G.P.; Tran, A.; Liu, D.; Butlin, M.; Avolio, A.; Boutcher, S.H.; Boutcher, Y.N. The Effect of Interval Sprinting Exercise on Vascular Function and Aerobic Fitness of Post‐menopausal Women. Scand. J. Med. Sci. Sports 2020, 30, 312–321, doi:10.1111/sms.13574.

S16. Jo, E.; Wu, S.; Han, H.; Park, J.; Park, S.; Cho, K. Effects of Exergaming in Postmenopausal Women with High Cardiovascular Risk: A Randomized Controlled Trial. Clinical Cardiology 2020, 43, 363–370, doi:10.1002/clc.23324.

S17. Jones, L.M.; Stoner, L.; Baldi, J.C.; McLaren, B. Circuit Resistance Training and Cardiovascular Health in Breast Cancer Survivors. Eur J Cancer Care 2020, 29, doi:10.1111/ecc.13231.

S18. Wong, A.; Figueroa, A.; Fischer, S.M.; Bagheri, R.; Park, S.-Y. The Effects of Mat Pilates Training on Vascular Function and Body Fatness in Obese Young Women with Elevated Blood Pressure. Am. J. Hypertens. 2020, 33, 563–569, doi:10.1093/ajh/hpaa026.

S19. Kirkman, D.L.; Ramick, M.G.; Muth, B.J.; Stock, J.M.; Pohlig, R.T.; Townsend, R.R.; Edwards, D.G. Effects of Aerobic Exercise on Vascular Function in Nondialysis Chronic Kidney Disease: A Randomized Controlled Trial. Am J Physiol Renal Physiol 2019, 316, F898–F905, doi:10.1152/ajprenal.00539.2018.

S20. Magalhães, J.P.; Melo, X.; Correia, I.R.; Ribeiro, R.T.; Raposo, J.; Dores, H.; Bicho, M.; Sardinha, L.B. Effects of Combined Training with Different Intensities on Vascular Health in Patients with Type 2 Diabetes: A 1-Year Randomized Controlled Trial. Cardiovasc. Diabetol. 2019, 18, 34, doi:10.1186/s12933-019-0840-2.

S21. Rahbar, S.; Naimi, S.S.; RezaSoltani, A.; Rahimi, A.; Baghban, A.A.; Noori, A.; Rashedi, V. Changes in Vascular Structure in Diabetic Patients after 8 Weeks Aerobic Physical Exercise: A Randomized Controlled Trial. Int. J. Diabetes Dev. Ctries. 2018, 38, 202–208, doi:10.1007/s13410-017-0579-9.

S22. Slivovskaja, I.; Ryliskyte, L.; Serpytis, P.; Navickas, R.; Badarienė, J.; Celutkiene, J.; Puronaite, R.; Ryliskiene, K.; Cypiene, A.; Rinkuniene, E.; et al. Aerobic Training Effect on Arterial Stiffness in Metabolic Syndrome. The American Journal of Medicine 2018, 131, 148–155, doi:10.1016/j.amjmed.2017.07.038.

S23. Azadpour, N.; Tartibian, B.; Koşar, Ş.N. Effects of Aerobic Exercise Training on ACE and ADRB2 Gene Expression, Plasma Angiotensin II Level, and Flow-Mediated Dilation: A Study on Obese Postmenopausal Women with Prehypertension. Menopause 2017, 24, 269–277, doi:10.1097/GME.0000000000000762.

S24. Bellia, A.; Iellamo, F.; De Carli, E.; Andreadi, A.; Padua, E.; Lombardo, M.; Annino, G.; Campoli, F.; Tartaglione, S.; D’Ottavio, S.; et al. Exercise Individualized by TRIMPi Method Reduces Arterial Stiffness in Early Onset Type 2 Diabetic Patients: A Randomized Controlled Trial with Aerobic Interval Training. Int. J. Cardiol. 2017, 248, 314–319, doi:10.1016/j.ijcard.2017.06.065.

S25. DeVallance, E.; Fournier, S.; Lemaster, K.; Moore, C.; Asano, S.; Bonner, D.; Donley, D.; Olfert, I.M.; Chantler, P.D. The Effects of Resistance Exercise Training on Arterial Stiffness in Metabolic Syndrome. Eur. J. Appl. Physiol. 2016, 116, 899–910, doi:10.1007/s00421-016-3348-4.

S26. Robinson, A.T.; Franklin, N.C.; Norkeviciute, E.; Bian, J.T.; Babana, J.C.; Szczurek, M.R.; Phillips, S.A. Improved Arterial Flow-Mediated Dilation after Exertion Involves Hydrogen Peroxide in Overweight and Obese Adults Following Aerobic Exercise Training. J. Hypertens. 2016, 34, 1309–1316, doi:10.1097/HJH.0000000000000946.

S27. Almenning, I.; Rieber-Mohn, A.; Lundgren, K.M.; Shetelig Løvvik, T.; Garnæs, K.K.; Moholdt, T. Effects of High Intensity Interval Training and Strength Training on Metabolic, Cardiovascular and Hormonal Outcomes in Women with Polycystic Ovary Syndrome: A Pilot Study. PLoS One 2015, 10, e0138793, doi:10.1371/journal.pone.0138793.

S28. Figueroa, A.; Kalfon, R.; Wong, A. Whole-Body Vibration Training Decreases Ankle Systolic Blood Pressure and Leg Arterial Stiffness in Obese Postmenopausal Women with High Blood Pressure. Menopause 2015, 22, 423–427, doi:10.1097/GME.0000000000000332.

S29. Figueroa, A.; Kalfon, R.; Madzima, T.A.; Wong, A. Whole-Body Vibration Exercise Training Reduces Arterial Stiffness in Postmenopausal Women with Prehypertension and Hypertension. Menopause 2014, 21, 131–136, doi:10.1097/GME.0b013e318294528c.

S30. Franklin, N.C.; Robinson, A.T.; Bian, J.-T.; Ali, M.M.; Norkeviciute, E.; McGinty, P.; Phillips, S.A. Circuit Resistance Training Attenuates Acute Exertion-Induced Reductions in Arterial Function but Not Inflammation in Obese Women. Metab Syndr Relat Disord 2015, 13, 227–234, doi:10.1089/met.2014.0135.

S31. Greenwood, S.A.; Koufaki, P.; Mercer, T.H.; Rush, R.; O’Connor, E.; Tuffnell, R.; Lindup, H.; Haggis, L.; Dew, T.; Abdulnassir, L.; et al. Aerobic or Resistance Training and Pulse Wave Velocity in Kidney Transplant Recipients: A 12-Week Pilot Randomized Controlled Trial (the Exercise in Renal Transplant [ExeRT] Trial). Am. J. Kidney Dis. 2015, 66, 689–698, doi:10.1053/j.ajkd.2015.06.016.

S32. Greenwood, S.A.; Koufaki, P.; Mercer, T.H.; MacLaughlin, H.L.; Rush, R.; Lindup, H.; O’Connor, E.; Jones, C.; Hendry, B.M.; Macdougall, I.C.; et al. Effect of Exercise Training on Estimated GFR, Vascular Health, and Cardiorespiratory Fitness in Patients with CKD: A Pilot Randomized Controlled Trial. Am. J. Kidney Dis. 2015, 65, 425–434, doi:10.1053/j.ajkd.2014.07.015.

S33. Oliveira, N.L.; Ribeiro, F.; Silva, G.; Alves, A.J.; Silva, N.; Guimarães, J.T.; Teixeira, M.; Oliveira, J. Effect of Exercise-Based Cardiac Rehabilitation on Arterial Stiffness and Inflammatory and Endothelial Dysfunction Biomarkers: A Randomized Controlled Trial of Myocardial Infarction Patients. Atherosclerosis 2015, 239, 150–157, doi:10.1016/j.atherosclerosis.2014.12.057.

S34. Van Craenenbroeck, A.H.; Van Craenenbroeck, E.M.; Van Ackeren, K.; Vrints, C.J.; Conraads, V.M.; Verpooten, G.A.; Kouidi, E.; Couttenye, M.M. Effect of Moderate Aerobic Exercise Training on Endothelial Function and Arterial Stiffness in CKD Stages 3-4: A Randomized Controlled Trial. Am. J. Kidney Dis. 2015, 66, 285–296, doi:10.1053/j.ajkd.2015.03.015.

S35. Croymans, D.; Krell, S.; Oh, C.; Katiraie, M.; Lam, C.; Harris, R.; Roberts, C. Effects of Resistance Training on Central Blood Pressure in Obese Young Men. J. Hum. Hypertens. 2014, 28, 157–164, doi:10.1038/jhh.2013.81.

S36. Donley, D.A.; Fournier, S.B.; Reger, B.L.; DeVallance, E.; Bonner, D.E.; Olfert, I.M.; Frisbee, J.C.; Chantler, P.D. Aerobic Exercise Training Reduces Arterial Stiffness in Metabolic Syndrome. J Appl Physiol (1985) 2014, 116, 1396–1404, doi:10.1152/japplphysiol.00151.2014.

S37. Headley, S.; Germain, M.; Wood, R.; Joubert, J.; Milch, C.; Evans, E.; Poindexter, A.; Cornelius, A.; Brewer, B.; Pescatello, L.S.; et al. Short-Term Aerobic Exercise and Vascular Function in CKD Stage 3: A Randomized Controlled Trial. Am. j. kidney dis. : off. j. Natl. Kidney Found. 2014, 64, 222–229, doi:10.1053/j.ajkd.2014.02.022.

S38. Pugh, C.J.A.; Sprung, V.S.; Kemp, G.J.; Richardson, P.; Shojaee-Moradie, F.; Umpleby, A.M.; Green, D.J.; Cable, N.T.; Jones, H.; Cuthbertson, D.J. Exercise Training Reverses Endothelial Dysfunction in Nonalcoholic Fatty Liver Disease. Am. J. Physiol. Heart Circ. Physiol. 2014, 307, H1298–H1306, doi:10.1152/ajpheart.00306.2014.

S39. Beck, D.T.; Casey, D.P.; Martin, J.S.; Emerson, B.D.; Braith, R.W. Exercise Training Improves Endothelial Function in Young Prehypertensives. Exp. biol. med. (Maywood N.J.) 2013, 238, 433–441, doi:10.1177/1535370213477600.

S40. Heydari, M.; Boutcher, Y.N.; Boutcher, S.H. High-Intensity Intermittent Exercise and Cardiovascular and Autonomic Function. Clin. Auton. Res. 2013, 23, 57–65, doi:10.1007/s10286-012-0179-1.

S41. Kadoglou, N.P.; Fotiadis, G.; Kapelouzou, A.; Kostakis, A.; Liapis, C.D.; Vrabas, I.S. The Differential Anti-Inflammatory Effects of Exercise Modalities and Their Association with Early Carotid Atherosclerosis Progression in Patients with Type 2 Diabetes. Diabet. Med. 2013, 30, e41-e50, doi:10.1111/dme.12055.

S42. Dobrosielski, D.A.; Gibbs, B.B.; Ouyang, P.; Bonekamp, S.; Clark, J.M.; Wang, N.-Y.; Silber, H.A.; Shapiro, E.P.; Stewart, K.J. Effect of Exercise on Blood Pressure in Type 2 Diabetes: A Randomized Controlled Trial. J GEN INTERN MED 2012, 27, 1453–1459, doi:10.1007/s11606-012-2103-8.

S43. Molmen-Hansen, H.E.; Stolen, T.; Tjonna, A.E.; Aamot, I.L.; Ekeberg, I.S.; Tyldum, G.A.; Wisloff, U.; Ingul, C.B.; Stoylen, A. Aerobic Interval Training Reduces Blood Pressure and Improves Myocardial Function in Hypertensive Patients. Eur. J. Prev. Cardiol. 2012, 19, 151–160, doi:10.1177/1741826711400512.

S44. Nualnim, N.; Parkhurst, K.; Dhindsa, M.; Tarumi, T.; Vavrek, J.; Tanaka, H. Effects of Swimming Training on Blood Pressure and Vascular Function in Adults >50 Years of Age. Am. J. Cardiol. 2012, 109, 1005–1010, doi:10.1016/j.amjcard.2011.11.029.

S45. Hermann, T.S.; Dall, C.H.; Christensen, S.B.; Goetze, J.P.; Prescott, E.; Gustafsson, F. Effect of High Intensity Exercise on Peak Oxygen Uptake and Endothelial Function in Long-Term Heart Transplant Recipients. Am. J. Transplant. 2011, 11, 536–541, doi:10.1111/j.1600-6143.2010.03403.x.

S46. Stensvold, D.; Tjønna, A.E.; Skaug, E.-A.; Aspenes, S.; Stølen, T.; Wisløff, U.; Slørdahl, S.A. Strength Training versus Aerobic Interval Training to Modify Risk Factors of Metabolic Syndrome. J. Appl. Physiol. 2010, 108, 804–810, doi:10.1152/japplphysiol.00996.2009.

S47. Loimaala, A.; Groundstroem, K.; Rinne, M.; Nenonen, A.; Huhtala, H.; Parkkari, J.; Vuori, I. Effect of Long-Term Endurance and Strength Training on Metabolic Control and Arterial Elasticity in Patients with Type 2 Diabetes Mellitus. Am. J. Cardiol. 2009, 103, 972–977, doi:10.1016/j.amjcard.2008.12.026.

S48. Loimaala, A.; Huikuri, H.V.; Kööbi, T.; Rinne, M.; Nenonen, A.; Vuori, I. Exercise Training Improves Baroreflex Sensitivity in Type 2 Diabetes. Diabetes 2003, 52, 1837-1842, doi:10.2337/diabetes.52.7.1837.

S49. Braith, R.W.; Schofield, R.S.; Hill, J.A.; Casey, D.P.; Pierce, G.L. Exercise Training Attenuates Progressive Decline in Brachial Artery Reactivity in Heart Transplant Recipients. J. Heart Lung Transplant. 2008, 27, 52-59, doi:10.1016/j.healun.2007.09.032.

S50. Bircher, S.; Mucha, C. Einfluss Körperlicher Aktivität Auf Die Fettoxidation Und Endothelfunktion Bei Adipösen - Eine Randomisiert Kontrollierte Studie. Phys. Med. Rehabil. Kurortmed. 2007, 17, 313–319, doi:10.1055/s-2007-973832.

S51. Olson, T.P.; Dengel, D.R.; Leon, A.S.; Schmitz, K.H. Moderate Resistance Training and Vascular Health in Overweight Women. Med. Sci. Sports Exercise 2006, 38, 1558–1564, doi:10.1249/01.mss.0000227540.58916.0e.
